# Taste and Safety: Is the Exceptional Cuisine Offered by High End Restaurants Paralleled by High Standards of Food Safety?

**DOI:** 10.1371/currents.outbreaks.007219ac3b9a2117418df7ab629686b6

**Published:** 2016-08-02

**Authors:** Sanch Kanagarajah, Piers Mook, Paul Crook, Adedoyin Awofisayo-Okuyelu, Noel McCarthy

**Affiliations:** Field Epidemiology Service, National Infection Service, Public Health England, London, United Kingdom; Field Epidemiology Service, National Infection Service, Public Health England, London, United Kingdom; Field Epidemiology Service, National Infection Service, Public Health England, London, United Kingdom; Gastrointestinal Infections, National Infection Service, Public Health England, London, United Kingdom; NIHR Health Protection Research Unit in Gastrointestinal Infections, United Kingdom; Field Epidemiology Service, National Infection Service, Public Health England, London, United Kingdom; NIHR Health Protection Research Unit in Gastrointestinal Infections, United Kingdom; Warwick Medical School, Division of Health Sciences, University of Warwick, Coventry, United Kingdom

**Keywords:** campylobacter, cooking, food poisoning, gastroenteritis, gourmet, norovirus, novel cooking, Outbreak

## Abstract

Introduction: Restaurant guides such as the Good Food Guide Top 50 create a hierarchy focussing on taste and sophistication. Safety is not explicitly included. We used restaurant associated outbreaks to assess evidence for safety.

Methods: All foodborne disease outbreaks in England reported to the national database from 2000 to 2014 were used to compare the Top 50 restaurants (2015) to other registered food businesses using the Public Health England (PHE) outbreak database. Health Protection Teams were also contacted to identify any outbreaks not reported to the national database. Among Good Food Guide Top 50 restaurants, regression analysis estimated the association between outbreak occurrence and position on the list.

Results: Four outbreaks were reported to the PHE national outbreak database among the Top 50 giving a rate 39 times higher (95% CI 14.5–103.2) than other registered food businesses. Eight outbreaks among the 44 English restaurants in the Top 50 were identified by direct contact with local Health Protection Teams. For every ten places higher ranked, Top 50 restaurants were 66% more likely to have an outbreak (Odds Ratio 1.66, 95% CI 0.89–3.13).

Discussion: Top 50 restaurants were substantially more likely to have had reported outbreaks from 2000-2014 than other food premises, and there was a trend for higher rating position to be associated with higher probability of reported outbreaks. Our findings, that eating at some of these restaurants may pose an increased risk to health compared to other dining out, raises the question of whether food guides should consider aspects of food safety alongside the clearly important complementary focus on taste and other aspects of the dining experience.

## INTRODUCTION

Restaurant guides such as the UK Good Food Guide Top 50 create a hierarchy focusing on taste and sophistication but not explicitly including consideration of safety^1^. Since restaurants are a common setting for outbreaks of gastrointestinal illness in the United Kingdom^2^, which can affect large numbers of diners^3^, food safety may also be an important criterion for some diners. The Food Standards Agency (FSA), in cooperation with local authorities, offers information on compliance with basic food safety standards across the catering industry through their public facing Food Hygiene Rating Scheme http://www.food.gov.uk/business-industry/caterers/hygieneratings. As a way to assess whether there is evidence of different levels of risk of foodborne disease among top restaurants we have reviewed restaurant associated outbreaks in England including those restaurants in the UK Good Food Guide Top 50.

## METHOD

Two complementary analyses were conducted. The first compared outbreaks in the Top 50 restaurants with outbreaks in all other restaurants in England using a national reporting database for foodborne disease outbreaks. The second tested whether position in the Top 50 was associated having an identified outbreak.

FSA data on registered food premises in 2014 were used to estimate the total number of food premises in England and the Good Food Guide Top 50 (2015) to identify the number of these 50 that were in England. The Public Health England (PHE) electronic Foodborne and non-Foodborne Gastrointestinal Outbreak Surveillance System (eFOSS), which is a national reporting system collating reports of outbreaks from PHE Health Protection Teams (HPT) and laboratory notifications, was used to identify outbreaks linked to food outlets from 2000 to 2014, and which of these was associated with restaurants listed in the UK Good Food Guide Top. PHE was formed in 2013 and it took on the role of the Health Protection Agency (2004-2013) which had taken on the functions of the national Communicable Disease Surveillance Centre. The function of eFOSS remained the same during these organisational transitions. The risk of outbreaks in the Top 50 was compared with the risk of outbreaks in other restaurants in England and rate ratios were calculated.

The 2015 edition of the UK Good Food Guide was also used to identify the position of individual English restaurants (excluding premises in Scotland and Wales) in the Top 50. HPTs in the areas where Top 50 restaurants in England were located were asked to identify outbreaks associated with these restaurants from 2000 to 2014 and to provide the following information on these outbreaks; the year of outbreak, organism identified, number of people affected, source of outbreak, agent, high risk food preparation processes, and the outbreak investigation report. The association between outbreak occurrence and position within the 2015 Top 50 was estimated using logistic regression analysis in this dataset. Possible risk factors identified in the investigation of the outbreaks were summarised.

Data were analysed with Stata version 13 and OpenEpi version 3.30a.

## RESULTS

FSA data identified 508,630 registered food premises in England in 2014. Forty four of the UK top 50 restaurants (88%) were located in England and included in this study, while the remaining restaurants were located in Wales (n=2) and Scotland (n=4). Among the 44 English restaurants identified in the Top 50, four had an outbreak reported to eFOSS (9.1%) compared to 1196 outbreaks reported from the other 508,586 registered food premises (0.23%) in England between 2000 and 2014; giving a Risk Ratio of 39 (95% CI 14.5-103.2).

Eight outbreaks in seven restaurants among the 2015 Top 50 were identified from 2000 to 2014 following direct contact with HPTs across England (Table 1). Among the 44 English Top 50 restaurants; a position higher on the ranking list was associated with a trend toward increased risk of an outbreak, with an odds ratio of 1.05 [0.99-1.12] for each place higher, equivalent to an odds ratio of 1.66 [0.89- 3.13] for being ten places higher in the list.

Of the seven restaurants in the Top 50 list in 2015 identified as having one or more outbreaks during the study period, five were in the top 50 list in the year that the outbreaks occurred (Table 1). Outbreak E occurred in a restaurant that opened in 2014, the same year as the outbreak, and so was not eligible for the Top 50 list for that year but was subsequently included in 2015. Outbreak H occurred in a restaurant that did not make the top 50 list in 2010 when it experienced an outbreak but was in the list from 2011 to 2015.

**Figure:**
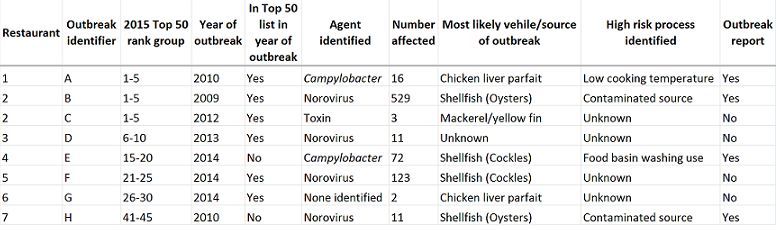
**Table 1.** Outbreaks in the 2015 Top 50 restaurants list reported in England from 2000-2014

**Descriptions of the outbreaks**

Outbreaks B and C occurred in the same restaurant (“restaurant 2”) in 2009 and 2012 respectively (Table 1). Outbreak reports were available for four out of the eight outbreaks and these are described in more detail.

Outbreak A was of Campylobacter associated in 2010. Sixteen cases were identified; 11 probable and five confirmed. Chicken liver parfait was determined to be the source but the organism was not identified via microbiological sampling from remaining food. The cooking method involved chicken livers soaked in milk, then pan fried with the temperature reaching 61 °C. The livers were then pureed in a heated blender with the temperature reaching 60 °C. The chicken livers were reportedly undercooked to avoid a grey appearance.

Outbreak B was an outbreak of norovirus reported as associated with the consumption of oysters. There were 529 reports of illness in January and February 2009. Ten stool samples were positive for norovirus. An analytical study identified one food, the ‘Oyster, Passion Fruit Jelly, Lavender’ from the ‘Tasting Menu’ to have the strongest association with illness. The lengthy period of transmission at the restaurant may have involved a combination of person to person spread among staff, contamination of pre-prepared foods, and persistent infection in seafood^3^.

Outbreak E was reported as an outbreak of norovirus associated with consumption of a plaice and cockle dish and subsequent contamination of other dishes in 2014. The first case was the chef followed by 39 staff members who reported being unwell during the first round of notifications. Seventy two cases were reported at the end of the outbreak with 15/20 stool samples testing positive for norovirus. Staff members who first became ill ate at a taster event. Although tweezers were used in part for food handling, environmental health officers identified substantial touching of food during preparation. They also noted that hand wash basins were also used for other purposes. An analytical study showed that cases were more likely to have eaten the plaice and cockles dish.

Outbreak H was reported as an outbreak of norovirus infection associated with the consumption of oysters in 2010. Eleven diners and one kitchen staff member were identified with symptoms of diarrhoea and vomiting. Environmental and stool samples tested positive for norovirus^4^. An analytical study showed an association of illness with oyster consumption.

## DISCUSSION

The Top 50 restaurants in England were more likely to have had reported outbreaks from 2000-2014 than other food premises, and there was a trend for higher rated position among the Top 50 to be associated with higher probability of reported outbreaks, although the latter findings were non-significant at the 5% level.

Limitations of this study which should be considered when interpreting these findings. Detected and reported outbreaks were the measure of food safety accessible to us across both the Top 50 restaurants and other registered food premises in England. Analyses identifying a 39 fold higher rate of reported outbreaks among Top 50 premises compared to other food premises and of higher position being associated with increased odds of an identified outbreak could each have been prone to bias in either direction. For example, if food premises within or further up the list in the Top 50 had a higher profile, and higher expectation from diners not to get sick, reporting and detection might be more likely. This could create an apparent positive association between high ranking and the occurrence of outbreaks. In contrast, if more prestigious restaurants use internal measures to respond to incidents without informing public health authorities^3^^,^ 4 unless very large or persistent outbreaks occur this could create a bias in the opposite direction. It is not clear whether systematic biases affect reporting of locally identified outbreaks to the national reporting system eFOSS. Direct contact with the local health protection teams revealed that four outbreaks occurring in top 50 restaurants had not been reported to the eFOSS system. Analysis of the association of position within the Top 50 and identified outbreaks showed a point estimate of 1.66 for every ten places higher in the ranking order. This may be less prone to bias but the numbers in this analysis are small and the non-significant trend identified may be due to chance alone. Due to lack of available data, these findings are unadjusted for potential confounders such as the size of the restaurant, the years of operation, the number of cases visiting the restaurants, or the number of dishes served at each restaurant. We used the 2015 version of the UK Good Food Guide and outbreaks from 2000 to 2014 so that the associations described may be best seen as whether having had outbreaks affected future presence and position in the rankings rather than position predicting risk of outbreaks. All but two restaurants were also in the top 50 list in the year of the outbreak. Due to the limitations in this study, several hypotheses and issues of bias remain to be tested in further studies: whether high end restaurant clientele are more likely to report illness, if the number of meals served in a restaurant may increase the number of cases, and if cooking methods used by the various restaurants can be investigated to see if certain methods cause more illness in diners in these settings.

A Food Standards Agency (FSA) Food Hygiene Rating Scheme is run by local authorities in England, Wales and Northern Ireland and applies to food businesses which are inspected by a food safety officer from the local authority. The score is calculated based on how hygienically the food is handled, the condition of the structure of the buildings and how the business manages and records what it does to make sure food is safe. The restaurants in the Top 50 were each awarded either 4 or 5 stars out of 5 on this FSA scheme during 2014. Despite scoring well on generic hygiene assessments there are a number of reasons as to why gourmet restaurants may have a higher rate of outbreaks. They may be more likely to serve foods with known risks such as raw oysters^5^; their complex dishes may require more handling and tasting by chefs; and novel and innovative cooking techniques may not robustly kill pathogens. There is some evidence for each of these factors from our study. Oysters or shell fish were identified as the most likely vehicle in four outbreaks, complex food processes were identified as a possible risk in two outbreaks^3^^,^ 4, and low cooking temperature of chicken liver parfait was identified in one outbreak. This range both overlaps and differs from findings of outbreaks in other settings as reported in the literature^6^^,^ 7 ^,^ 8 ^,^ 9. Similarities with wider studies on outbreaks includes the importance of lightly cooked chicken liver products prepared in restaurants that have been identified as an important source of gastroenteritis outbreaks in recent years 10. Differences include the lack of Salmonella compared to an England and Wales study that where these accounted for most outbreaks, particularly among Chinese, Indian, British and Italian cuisines from 1992 to 2009^11^. The pathogen profile and risk processes also differ from those reported from domestic premises which were associated with Salmonella, inappropriate storage of food, and consumption of poultry, eggs, or sauces^12^. There is thus some evidence for differences in the types of food safety risks that predominate in high end restaurants compared to other catered settings.

Overall the eight outbreaks identified at restaurants rated as in the top 50 in the country by the Good Food Guide affected more than 750 diners. Our findings, that eating at some of these restaurants may pose an increased risk to health compared to dining out at other establishments, raises the question of whether food guides should consider aspects of food safety alongside the clearly important complementary focus on taste and other aspects of the dining experience. Our study also gives some oversight into the range of foods that may contribute to the risk of outbreaks in these high end restaurants and may offer a steer to the more cautious diner’s selections in these settings.

## Competing Interest

The authors have declared that no competing interests exist.
